# Cardiac Arrest with Multi-vessel Coronary Artery Disease and Successful Treatment After Long Conventional Cardiopulmonary Resuscitation: How Long Is Too Long?

**DOI:** 10.7759/cureus.5993

**Published:** 2019-10-25

**Authors:** Rashid Nadeem, Hesham Osman, Yehia Karaly, Ibrahim AlBakri, Fayaz Khazi

**Affiliations:** 1 Intensive Care Medicine, Dubai Hospital, Dubai, ARE; 2 Interventional Cardiology, Dubai Hospital, Dubai, ARE; 3 Cardiac Anesthesiology, Dubai Hospital, Dubai, ARE; 4 Cardiology, Dubai Hospital, Dubai, ARE; 5 Cardiothoracic Anesthesia, Dubai Hospital, Dubai, ARE

**Keywords:** cardiopulmonary resuscitation, coronary artery disease, per cutaneous intervention, cardiac bypass, coronary stenting

## Abstract

Coronary artery disease (CAD) is the most common killer disease, responsible for about one-third of all deaths at ages above 35. The majority of all survivors of out-of-hospital cardiac arrests present to the emergency department (ED) with an initial shockable rhythm (ventricular fibrillation or pulse-less ventricular tachycardia), which is a predictor of survival. Odds for survival are less for non-shockable rhythm and favorable neurologic outcomes decrease as the length of cardiopulmonary resuscitation (CPR) increases. The median time-to-return of spontaneous circulation among those with favorable neurological outcomes is less than 10 minutes. On the other hand, a large review of more than 64,000 patients with in-hospital cardiac arrests showed that patients with longer median resuscitation times had a greater chance of the return of spontaneous circulation and survival to discharge. We described a case of prolonged resuscitation lasting almost three hours of CPR followed by successful percutaneous intervention with a favorable neurologic outcome.

## Introduction

Coronary artery disease (CAD) is the most common killer disease [1]. The presentation of patients with CAD range from simple chest pain or angina in the office setting to presenting as severe chest pain with new-onset electrocardiogram (EKG) changes - ST elevation.

Most patients who survived presented to the emergency department with arrhythmia-like ventricular fibrillation (VF) or pulseless ventricular tachycardia (VT), which are shockable rhythms with a good prognosis [2]. Presentation with rhythms other than VF/VT are treated without defibrillation as per the advanced cardiac life support (ACLS) protocol and are suggestive of a less favorable prognosis. For out-of-hospital arrests, the odds of survival decrease as the duration of cardiopulmonary resuscitation (CPR) increases. In addition, the longer the CPR, the more the loss of neurologic function, as the quality of CPR is a major determinant of circulation and perfusion to the brain. A return of spontaneous circulation (ROSC) within 10 minutes suggests favorable neurological outcomes [3-4].

In-hospital cardiac arrest in contrast to out of hospital cardiac arrest has a different course and prognosis. A large review of more than 64,000 patients with in-hospital cardiac arrests showed that patients with longer median resuscitation times had a greater chance of ROSC and better survival to discharge [5]. The quality of chest compression is critical for maintaining circulation. There is no consensus about how long is enough, especially in the case of extracorporeal membrane oxygenation.

We describe a case of prolonged CPR lasting almost three hours, followed by a successful percutaneous coronary intervention (PCI) with a favorable neurologic outcome.

## Case presentation

**Figure 1:**
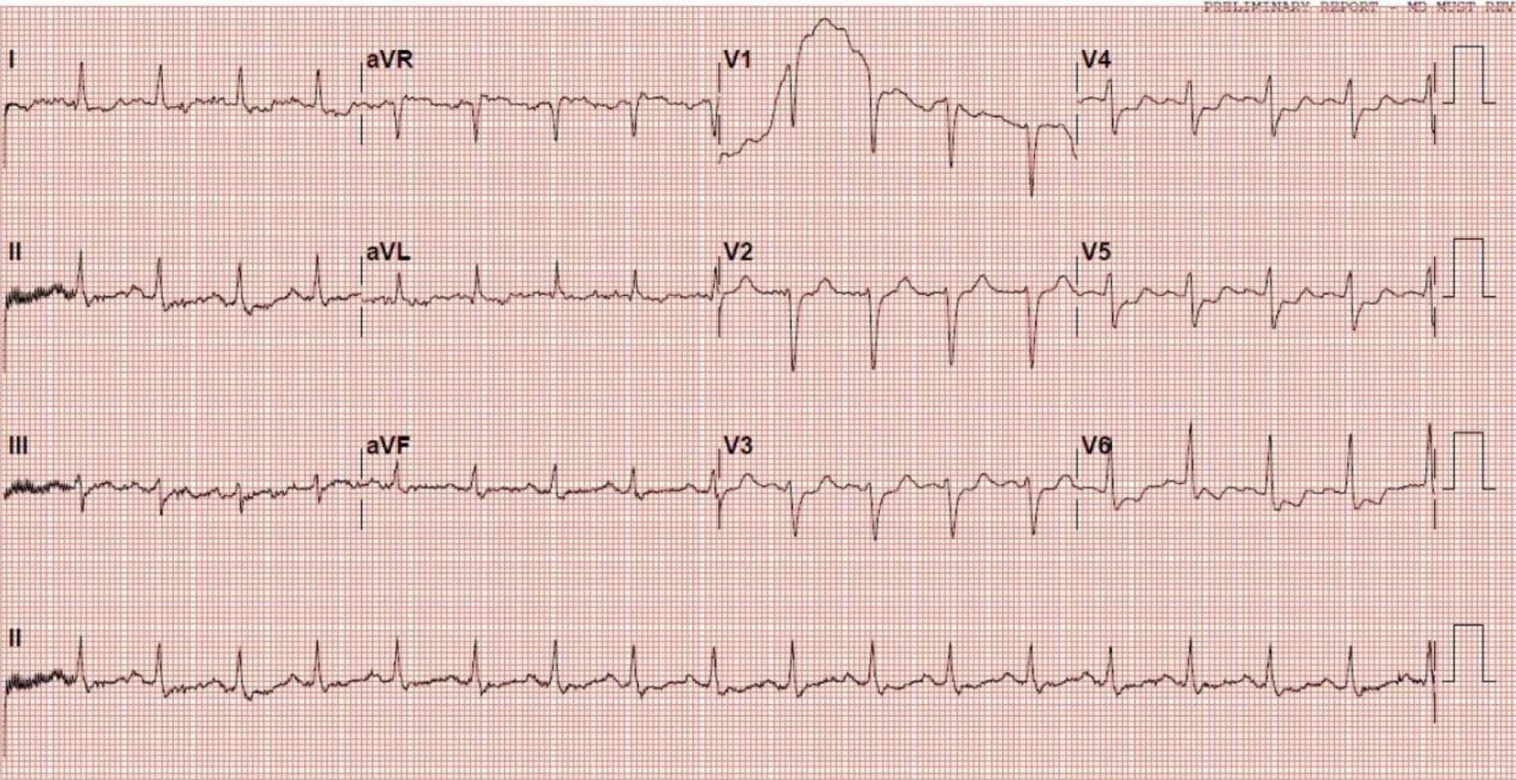
EKG at presentation EKG: electrocardiogram

A 64-year-old female, with a history of type 2 diabetes mellitus, essential hypertension, dyslipidemia, CAD, and angioplasty performed 16 months ago for right coronary artery (RCA) and left anterior descending (LAD) lesions, presented to the emergency department (ED) with decompensated heart failure. The patient was taking the following medication at home: aspirin, clopidogrel, bisoprolol, atorvastatin, spironolactone, lasix, rabeprazole, vildagliptin-metformin, gliclazide, and glargine insulin. On admission, laboratory tests were complete blood count (CBC): hemoglobin 13.7 mg/dl, platelet 152,000, and white blood cell (WBC) 8000; normal renal functions, electrocardiogram (EKG) with no ST-elevation (Figure 1), and echo showed an ejection fraction of 45%, with inferior and inferolateral wall motion abnormalities.

**Figure 2:**
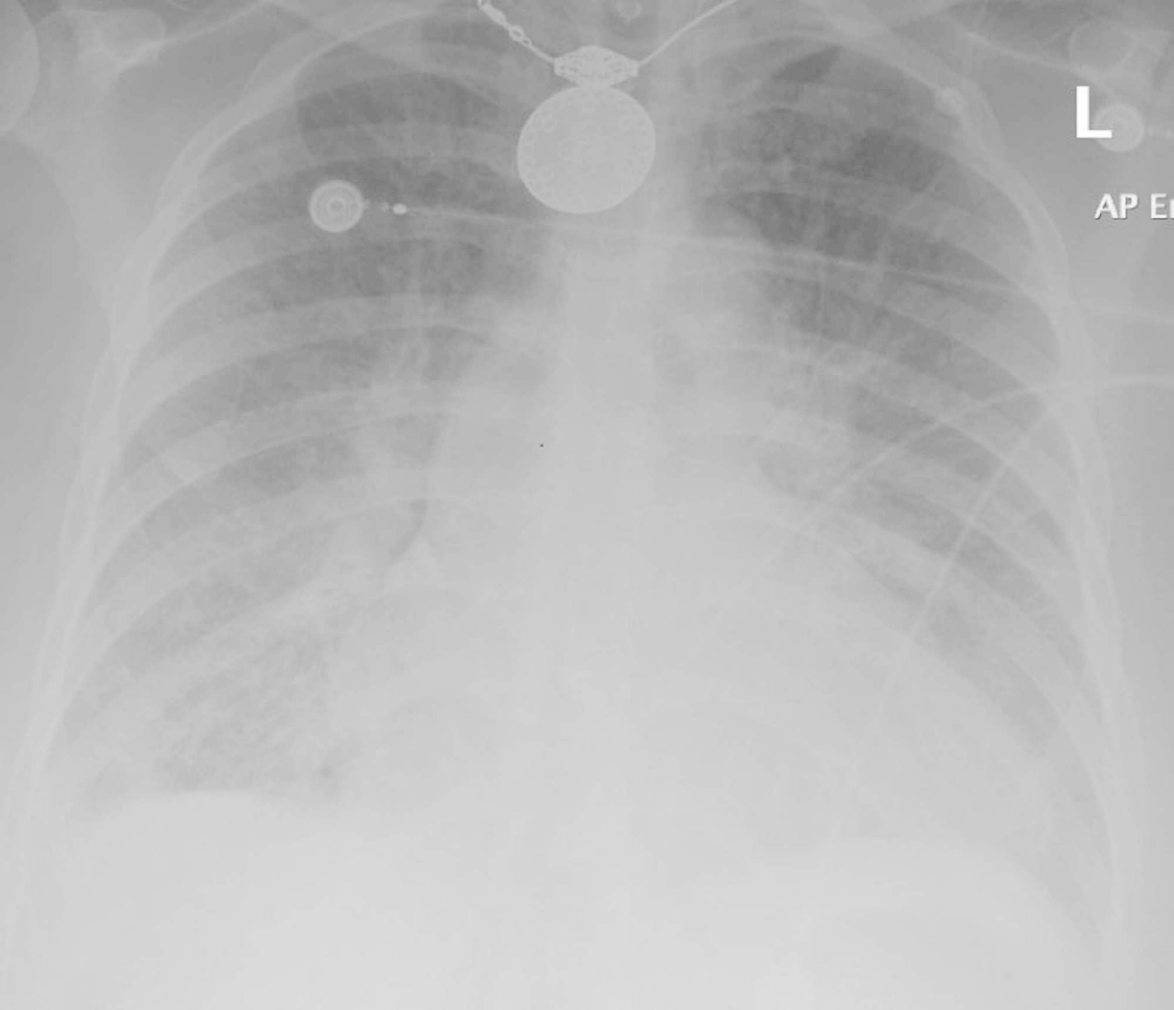
Chest X-ray showing pulmonary edema

The patient underwent a coronary angiogram and was found to have a critical multi-vessel disease. She was transferred to our hospital for coronary artery bypass surgery (CABG) planned for the next day. A few hours after arrival at our hospital, the patient suddenly developed pulmonary edema (Figure 2) and cardiac arrest requiring CPR and mechanical ventilation.

The patient had pulseless electrical activity (PEA). The ACLS protocol was followed for about every 20 minutes or so. The patient showed some morphological changes in rhythm while being pulseless, so high-quality CPR (minimizing interruptions in chest compressions, providing compressions of adequate rate and depth, avoiding leaning on the chest between compressions, and avoiding excessive ventilation) was continued, initially, manually by physicians and, later, with the help of Lucas-assisted CPR. The patient gained ROSC after 160 minutes of CPR. While transferring to the operation theater for emergent CABG, the patient exhibited upper gastrointestinal (GI) bleeding so an urgent gastroscopy at the bedside was performed, as the patient was to be subjected to anticoagulation for a cardiac bypass. The patient was found to not have any significant bleeding and she required no intervention. After a multidisciplinary meeting, including cardiac surgery, cardiology, and her family, a decision was made to proceed with PCI instead since the patient was at high risk for bleeding with heparin and had a prolonged cardiac arrest. Moreover, the gastroscopy picture showed no significant bleeding, and it suggested that the patient will be able to use antiplatelet therapy, which is required in every patient having PCI with stenting. The patient underwent PCI, 90% stenosis of LAD (Video 1), and 90% stenosis of RCA were treated with angioplasty followed by the placement of a drug-eluting coronary stent (Video 2). The next day, sedatives were held and the patient underwent a neurological assessment. The patient was able to move all extremities on stopping sedation. The following day, the patient was successfully extubated, as the ventilator was not required anymore. The patient had good neurologic functions and memory and was discharged home the following day with outpatient follow-up in the cardiology clinic.

**Video 1:** LAD lesion before angioplasty LAD: Left anterior descending

**Video 2:** Angiogram post LAD stenting LAD: Left anterior descending

## Discussion

Cardiac arrest from coronary artery disease is a common complication. The timely start of good-quality CPR is critical to prevent any anoxic brain damage. Studies show time to ROSC is a predictor of neurologic injury though there is no consensus about what length of CPR is adequate. Survival to discharge, especially with a good neurological outcome, decreases with a longer CPR duration for out-of-hospital cardiac arrests [6]. Recently, extracorporeal membrane oxygenation (ECMO) is gaining popularity in the resuscitation of patients with severe cardiopulmonary compromise [7]. There is no consensus on how long CPR should be continued in a center where ECMO is not an option as the bridge to definitive treatment. There are many cases reported for patients with CPR for more than 100 minutes [3]. In our case, CPR, lasted 160 minutes, and the patient had excellent neurologic outcomes. Our case highlights the importance of the clinical judgment of the CPR providers. It also reaffirms that high-quality CPR is critical to clinical outcomes regardless of the duration of CPR.

After ROSC, the uncertainty of neurologic status or complications like gastrointestinal bleeding may affect the subsequent management of CABG versus PCI. There is no guideline regarding the timing of definitive treatment intervention. The assessment of neurologic status may delay definitive treatment and may result in the recurrence of cardiac arrest. Most clinicians would agree that immediate PCI would be more appropriate in our patient, instead of waiting to observe or immediate CABG. A thorough multidisciplinary approach, including the family to discuss risks and benefits, is essential to make an informed, optimal management decision. Success stories like this one help clinicians to be optimistic and observant of any clinical signs suggestive of continuing resuscitative efforts. Good quality CPR is the key to success.

## Conclusions

Good clinical judgment should always be exercised in every patient with a cardiac arrest who has a potentially reversible cause, regardless of the duration of CPR. Dynamic changes during CPR are suggestive of a potentially modifiable process, which may provide a basis to continue good quality CPR. Most centers consider ECMO early if the patient is in cardiogenic shock, to protect other organ systems from acute decompensation. In our patient, we monitored the quality of CPR by electronic feedback from the device itself in addition to periodic monitoring by echo and Doppler to assess cardiac function. We believe our case is an exception rather than the routine practice in most centers.
